# Dynamic residential movement and depression among the World Trade Center Health Registry enrollees

**DOI:** 10.1007/s00127-021-02192-9

**Published:** 2021-11-16

**Authors:** Sungwoo Lim, Sze Yan Liu, Jennifer Brite, Aldo Crossa, Sean Locke, Cristina Pollari, María Baquero

**Affiliations:** 1 Division of Epidemiology, Bureau of Epidemiology Services, New York City Department of Health and Mental Hygiene, 42-09 28th Street, Long Island City, NY 11101 USA; 2 Montclair State University, Montclair, NJ USA; 3 York College of the City University of New York, Jamaica, NY USA; 4 Division of Epidemiology, Bureau of World Trade Center Registry, New York City Department of Health and Mental Hygiene, Long Island City, NY USA

**Keywords:** Housing, Depression, Social environment

## Abstract

**Purpose:**

Residential instability is associated with poor mental health, but its causal inference is challenging due to time-varying exposure and confounding, and the role of changing social environments. We tested the association between frequent residential moving and depression risk among adults exposed to the 9/11 disaster.

**Methods:**

We used four waves of survey data from the World Trade Center Health Registry. We measured residential movement and depression using geocoded annual address records and the Personal Health Questionnaire Depression Scale, respectively, for a prospective cohort of 38,495 adults. We used the longitudinal Targeted Maximum Likelihood Method to estimate depression risk by frequent residential moving and conducted causal mediation analysis to evaluate a mediating role of social environments.

**Results:**

Most enrollees (68%) did not move in 2007–2014, and 6% moved at least once every 4 years. The remaining 26% moved less frequently (e.g., only moving in 2007–2010). Frequent moving versus no moving was associated with risk of depression in 2015–16 (RR = 1.20, 95% CI = 1.06, 1.37). Frequent residential moving—depression pathway was mediated by high social integration (OR = 0.93, 95% CI = 0.90, 0.97).

**Conclusion:**

These findings demonstrate the importance of social networks in understanding increased risk of depression associated with housing instability.

## Introduction

Housing stability is an important social determinant of health, directly and indirectly shaping an individual's health across their life course. Negative health impacts of frequent residential moving have been observed, especially among children and adolescents, as life disruption due to moving during a sensitive and critical development period may adversely impact their ability to handle stress. According to a large Swedish study, residential moving during childhood was associated with higher risk of nonaffective psychosis [1]. The authors found a dose–response relationship between number of residential moves and risk of nonaffective psychosis among these children. A study in the United States also showed that moving during adolescence was associated with depressive symptoms, and this association was influenced by social support from teachers and others [2].

However, unlike children and adolescents, the association between frequent residential moving and health has been less clear among adults. For example, among adult drug users in Baltimore, frequent moving was associated with depressive symptoms [3]. However, frequent moving among adults in Michigan was not associated with poor mental health outcomes unless they moved due to cost concerns after the 2008 financial crisis [4].

These mixed findings could reflect complex social and economic contexts and motivations of moving among adults compared with children whose moves tend to be involuntary. As pointed out in a recent study, it may be critical to take into account changes in social environments resulting from frequent moving (e.g., loss of social cohesion and social support) when assessing complex relationships between frequent residential moving and health [5]. Yet, there is little research on social environments as a mediator of the frequent moving—health pathway. Another explanation for the mixed findings might be that key causal assumptions could be violated due to time-varying exposure and confounding. For example, the assumption that a treatment effect is homogeneous across all levels of confounders is highly untenable when confounding is time-varying [6]. When exposure itself is time-varying such as frequent residential moving, bias from time-varying confounding cannot be ruled out in a causal estimate of housing instability and health association. A special causal inference technique such as Targeted Maximum Likelihood Estimation (TMLE) is required to draw a valid causal inference from longitudinal data with time-varying exposure and confounding [7].

To address these gaps in the current literature, we aimed to answer the following research questions. First, was moving frequently over eight years associated with higher risk of depression among adults exposed to the 9/11 disaster? Second, was the frequent residential moving—depression pathway explained by social environments? To answer these questions, we used a rich longitudinal dataset of people exposed to the 9/11 disaster and made inference via TMLE to address violations of the casual assumptions.

## Materials and methods

### Population and data sources

Data for this prospective cohort study came from the World Trade Center Health Registry (WTCHR), a longitudinal cohort study of individuals exposed to the World Trade Center (WTC) terrorist attacks on September 11, 2001. Those eligible for the Registry include people who lived, worked, or went to school in lower Manhattan, passers-by, and rescue/recovery workers and volunteers. About 90% of the adult registry enrollees lived in New York City (65%), other areas in New York State (15%), and New Jersey (10%) on September 11, 2011 [8]. Of these, a large number of New York City residents came from lower Manhattan. The Registry has followed the adult registry enrollees over 12 years and conducted 4 periodic surveys in 2003–2004 (Wave 1), 2006–2007 (Wave 2), 2010–2011 (Wave 3), and 2015–2016 (Wave 4) via web, paper, or computer-assisted telephone interviews. Detailed descriptions of WTCHR recruitment and data collection are described elsewhere [8].

The present analysis is based on a subset of adult Registry enrollees who participated in both Wave 1 and Wave 2 and had non-missing data at Wave 2 on 5 time-varying potential confounders (age, post-traumatic stress disorder (PTSD), current smoking, marital status, and employment). Additional inclusion criterion was non-missing annual Public Use Microdata Areas (PUMA) of residence data, which were derived from geocoded address data. These resulted in a final sample of 38,495 Registry enrollees. Of these, 10,302 and 7047 enrollees were dropped out at Waves 3 and 4, respectively. These enrollees were more likely to be non-Latino Black or Latino, currently smoke, and report lower household incomes in 2002, and less likely to be married than those who remained in the Waves 3 and 4 surveys. On the other hand, there was no systematic difference by attribution in terms of Wave 1 education and PTSD.

The Institutional Review Boards of the Centers for Disease Control and Prevention and New York City Department of Health and Mental Hygiene (IRB protocol #: 02-058) approved the study protocol.

### Variables

The primary outcome variable for this study was current depression at Wave 4, defined as a score ≥ 10 according to the Personal Health Questionnaire Depression Scale. This scale was first included at Wave 3 and self-administrated without clinicians’ review. However, validity of this measure has been well established [9]. The time-varying exposure variable was residential movement at the 4-year period between Wave 2 and Wave 3 (2007–2010) and the 4-year period between Wave 3 and Wave 4 (2011–2014). Any year-by-year change of PUMA of residence during each of two time points (2007–2010 and 2011–2014) was classified as residential movement and summarized using 4 time-varying exposure scenarios: Stay–Stay, Move–Move, Move–Stay, and Stay–Move. In this study, we did not consider local movements within the existing neighborhood as residential movement because changes in social environments associated with local movements, which is one of our main proposed pathways, were quite unlikely. Because the primary study outcome, current depression, started to be collected at Wave 3, and the interval between Wave 1 and Wave 2 were shorter than those between subsequent waves (2 years vs. 4 years on average), we did not take into account residential movement between Waves 1 and 2 in the exposure variable. In addition to the outcome and exposure variables, we included covariates that were identified as common causes or intermediary variables of the frequent moving and depression pathway according to a Directed Acyclic Graphic (Fig. 2). Potential Wave 1 confounders included demographics (sex, race/ethnicity, education, household income) and 9/11 disaster-related characteristics (serving as uniformed workers during the 9/11 disaster, serving as rescue/recovery/cleanup workers at World Trade Center, Staten Island, and/or barge sites, and developing probable PTSD according to the civilian version of the PTSD checklist) [10, 11]. Time-varying variables at Waves 2 and 3 included age, current smoking, marital status, employment status, and PTSD. To account for intermediary paths through changes in the outcome, we included a measure of current depression at Wave 3. Lastly, to explore probable explanations for an association between frequent moving and depression, we included unmet medical and mental health needs and social integration at Waves 3 and 4. Specifically, unmet healthcare needs were measured by asking if a person did not receive needed healthcare or counseling in the past 12 months. Social integration was measured using the 4-item RAND Social Health Battery and categorized into low/medium vs. high scores [12]. We addressed missing data at Waves 3 (*n* = 10,302) and 4 (*n* = 7047) by incorporating censoring models in TMLE (described in detail in the statistical analyses section).

### Statistical analysis

We drew causal inference using TMLE. Causation is established when the outcome under the actual exposure (e.g., Move–Move) is different from the outcome under the counterfactual exposure in the same individual (e.g., Stay–Stay). Counterfactual outcomes (e.g., depression for a frequently moving individual under the hypothetical condition that residential movement did not occur) are unobserved and in TMLE these are considered missing data and substituted with predicted values via modeling [7]. Specifically, the outcome model for depression was constructed using time-invariant covariates, a history of the outcome (current depression at Wave 3), exposure (residential movement in 2007–2010, residential movement in 2011–2014), and time-varying covariates, which in turn produced conditional expectations of depression under the actual residential movement condition as well as counterfactuals for each person. Two additional models for residential movement (propensity score) and loss to follow-up (censoring) were constructed using the same set of covariates from the outcome model. Likelihood of receiving an exposure (propensity score; moving vs. staying) and likelihood of being lost to follow-up (censoring) were explicitly estimated and then incorporated values of the outcome variable predicted by the outcome model. This process helps address (1) bias due to differences in demographics and 9–11 disaster-related characteristics by moving vs. staying (propensity score), and (2) bias due to missing data (censoring). In addition, use of the same set of variables in the outcome, propensity, and censoring models helps reduce bias due to misspecification. Starting from the last time point of the follow-up, these three types of modeling processes were repeated for each time point in a backward order, which is known as a recursive conditional likelihood method [13, 14]. Updated conditional expectations by exposure status via recursive conditional likelihood were compared and averaged over all study subjects, yielding a causal risk ratio. Inference was made by the efficient influence curve equation.

TMLE for the frequent residential moving—depression pathway was estimated under 3 different scenarios (scenario #1: Move–Move, scenario #2: Move–Stay, scenario #3: Stay–Move) against a counterfactual pattern (Stay–Stay). Estimation was made using the machine learning approach via Superlearner [15]. Specifically, it uses a data adaptive algorithm whereby a series of estimators were calculated via various methods such as random forest, elastic net, regression trees, and generalized additive modeling, and the best weighted combination of estimators are selected via cross-validation, which could address bias due to model misspecification and other violations of statistical assumptions.

To test whether or not social environments mediate a relationship between frequent residential moving and depression, we used two additional modeling approaches because causal mediation analysis was not developed for time-varying exposure and outcome within the TMLE framework. First, we replaced depression with each of 3 potential mediators as the outcome (unmet medical needs, unmet mental health needs, and high social integration) and repeated TMLE analyses to test the association between frequent residential moving and each outcome. Since these analyses only tested a pathway between exposure and mediator, second, we performed causal mediation analyses using the complete data from Wave 4 participants. Specifically, we replaced time-varying variables with time-invariant variables and tested if each of 3 potential mediators mediated the association between frequent moving and depression at Wave 3 [16]. We estimated both natural direct and indirect effects using odds ratios to follow the mediation formula for binary outcomes [17].

TMLE analyses and causal mediation analyses were conducted using R software LTMLE package, and medflex package, respectively [17, 18]. All other analyses were performed using SAS 9.4 software (SAS Institute, Inc., Cary, NC). Statistical significance was determined by a 2-sided *p*-value < 0.05.

## Results

A majority of 36,464 WTCHR enrollees (68%) did not move during 2007 to 2014 (Stay–Stay). The remaining 32% moved at least once during the 8-year period. Specifically, 6% of the enrollees moved at least once during 2007–2010 as well as during 2011–14 (Move–Move), while 14% moved only during the first 4-year period (Move–Stay) and 12% only moved during the second 4-year period (Stay–Move). As seen in Table 1, socioeconomic status, race/ethnicity, and prevalence of PTSD at Wave 1 were similar across the 4 residential moving patterns. However, those with Move–Move vs. Stay–Stay were younger (average 38 years old vs. 46 years old at Wave 1) and less likely to be a uniformed service member (8% vs. 17% at Wave 1). The percent of those who were married increased between Waves 2 and 3 among enrollees who moved at least once over 8 years (Table 2), while there was no change among those who did not move. Among those with the Move–Move pattern, there was a smaller decrease in the percent of those who were employed between Waves 2 and 3. There was little variation in the other time-varying variables across residential moving patterns.

**Table 1:** Baseline demographic, socioeconomic, and 9/11-related characteristics by residential moving patterns among World Trade Center Health Registry Survey Participants, New York, 2003–2016

	All	Stay–Stay	Move–Move	Move–Stay	Stay–Move
*N* (%)	38,464	26,319 (68%)	2290 (6%)	5243 (14%)	4612 (12%)
Demographic, socioeconomic characteristics					
Income in 2002					
< $10,000	3.2%	2.7%	6.0%	2.7%	4.7%
$10,000– < $15,000	1.9%	1.8%	2.5%	1.5%	2.2%
$15,000– < $25,000	3.5%	3.4%	4.4%	3.1%	4.2%
$25,000– < -$50,000	18.9%	17.4%	24.0%	19.9%	23.8%
$50,000– < $75,000	21.7%	21.5%	23.1%	23.0%	20.9%
$75,000– < $150,000	37.8%	40.0%	28.7%	36.6%	31.3%
$150,000 +	13.1%	13.3%	11.4%	13.2%	12.8%
Education					
< high school degree	3.2%	3.6%	1.9%	2.0%	3.4%
High school degree	18.3%	19.5%	14.2%	14.8%	17.2%
Some college +	78.3%	76.7%	83.7%	83.0%	79.2%
Missing	0.3%	0.3%	0.1%	0.2%	0.2%
Race/ethnicity					
Non-Latino White	70.6%	71.0%	70.9%	71.9%	66.9%
Non-Latino Black	10.0%	10.3%	8.4%	8.8%	10.8%
Latino	11.2%	10.6%	12.4%	12.0%	13.5%
Asian	5.3%	5.3%	5.7%	4.6%	5.8%
Others	2.8%	2.9%	2.6%	2.7%	2.9%
Average age in years at Wave 1	44	46	38	41	41
9/11-related characteristics					
Uniformed service member	15.5%	16.6%	8.4%	14.8%	13.5%
Rescue, recovery, cleanup worker	46.4%	47.4%	41.5%	45.9%	43.4%
PTSD at Wave 1					
Yes	14.0%	13.8%	14.7%	14.7%	14.4%
Missing	2.2%	2.4%	1.3%	1.6%	2.2%

Missing income data were imputed using demographic, clinical, behavioral, and social characteristics

**Table 2:** Time-varying characteristics by residential moving patterns among World Trade Center Health Registry Survey Participants, New York, 2003–2016

	All	Stay–Stay	Move–Move	Move–Stay	Stay–Move
*N*	38,464	26,319	2290	5243	4612
PTSD at Wave 2	20%	19%	21%	20%	21%
PTSD at Wave 3	16%	15%	18%	17%	17%
Average ages in years at Wave 2	47	49	41	44	44
Average ages in years at Wave 3	52	54	45	49	50
Being married at Wave 2	69%	71%	58%	69%	61%
Being married at Wave 3	70%	71%	62%	72%	63%
Current smoking at Wave 2	14%	13%	17%	13%	17%
Current smoking at Wave 3	10%	10%	13%	11%	14%
Being employed at Wave 2	82%	81%	81%	81%	84%
Being employed at Wave 3	71%	71%	75%	73%	71%

Missing data were not included in the denominator

**Fig. 1:**
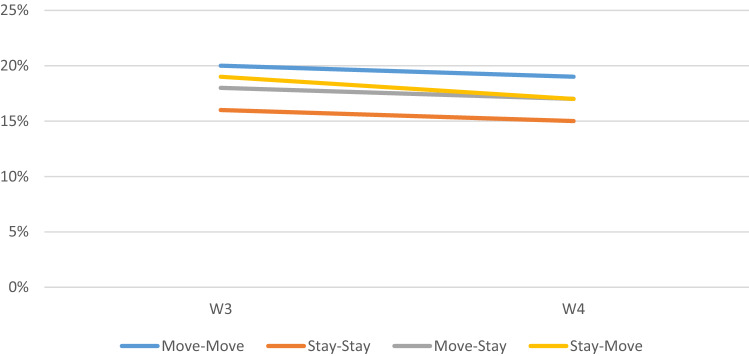
Crude Prevalence of Depression by Residential Moving Patterns Among World Trade Center Health Registry Survey Participants, New York, 2003–2016

**Fig. 2:**
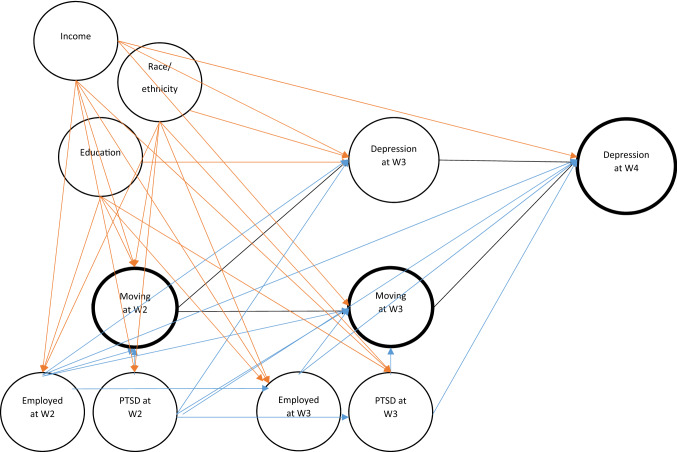
Directed Acyclic Graph^a^. ^a^Simplified for the illustration purpose

Figure 1 shows that prevalence of current depression at Wave 3 ranged from 16% (Stay–Stay) to 20% (Move–Move). After 4 years, these estimates slightly decreased to 15% (Stay–Stay) and 19% (Move–Move), and the trend was almost parallel across all residential moving patterns.

According to the TMLE, frequent moving (Move–Move) versus no moving (Stay–Stay, reference) was associated with 1.2 times higher risk of depression at Wave 4 (95% Confidence Interval (CI) = 1.06, 1.37, Table 3). On the other hand, other movement patterns versus the reference were not associated with risk of depression at Wave 4. Risk ratio of depression for two other residential moving patterns versus the reference was not statistically significant (Move–Stay vs. Stay–Stay: RR = 1.05, 95% CI = 0.96, 1.14; Stay–Move vs. Stay–Stay: RR = 0.99, 95% CI = 0.90, 1.08).

**Table 3:** Association between depression and residential moving patterns among World Trade Center Health Registry Survey Participants, New York, 2003–2016

Residential moving patterns	RR of depression^a^ (95% CI^a^)	*P*-value
Stay–Stay (reference)	–	–
Move–Move	1.20 (1.06, 1.37)	< 0.01
Move–Stay	1.05 (0.96, 1.14)	0.28
Stay–Move	0.99 (0.90, 1.08)	0.80

*CI* confidence interval, *RR* risk ratio

^a^Estimated via Longitudinal Targeted Maximum Likelihood Estimation with baseline and time-varying covariates. Baseline covariates included sex, race/ethnicity, education, household income, serving as uniformed workers during the 9/11 disaster, serving as rescue/recovery/cleanup workers at World Trade Center, Staten Island, and/or barge sites, and developing probable PTSD. Time-varying covariates included age, current smoking, marital status, employment status, and PTSD at Waves 2 and 3. In addition, current depression at Wave 3 was included to account for intermediary paths through changes in the outcome

To understand potential mechanisms of the residential moving–depression pathway, we examined differences in unmet health needs and social integration by residential moving patterns. While unmet physical health needs substantially decreased from Wave 3 to Wave 4, unmet mental health needs increased and social integration did not change much during the same time period (Table 6). According to the TMLE analysis (model 1), enrollees with Move–Move pattern were less likely to experience high social integration at Wave 4, compared with those with Stay–Stay pattern (RR = 0.91, 95% CI = 0.85–0.97, Table 4). According to the causal mediation analysis for the Wave 4 participants with the complete data (model 2), enrollees were less likely to experience depression if their residential moving patterns were fixed at Move–Move pattern, while changing the level of social integration to the level of the enrollees in the Stay–Stay pattern (OR for natural indirect effect = 0.93, 95% CI = 0.90–0.97, Table 5). A natural direct effect for Move–Move vs. Stay–Stay patterns via social integration was also statistically significant, indicating that frequent residential moving was associated with depression if social integration was fixed at the level that would be observed if participants followed the Stay–Stay pattern (OR = 1.22, 95% CI = 1.02, 1.47). On the other hand, there were no significant natural direct and indirect effects of frequent residential moving on depression via unmet mental and physical health needs.

**Table 4:** Association between Unmet Health Needs/Social Integration and Residential Moving Patterns Among World Trade Center Health Registry Survey Participants, New York, 2003–2016

Residential moving patterns	High social integration	Unmet physical health needs	Unmet mental health needs
	RR^a^ (95% CI^a^)	RR^a^ (95% CI^a^)	RR^a^ (95% CI^a^)
Stay–Stay (reference)	–	–	–
Move–Move	0.91 (0.85, 0.97)	1.01 (0.61, 1.67)	1.07 (0.83, 1.39)
Move–Stay	0.96 (0.96, 1.00)	0.97 (0.71, 1.31)	0.86 (0.72, 1.02)
Stay–Move	0.99 (0.95, 1.03)	1.09 (0.79, 1.51)	1.06 (0.88, 1.27)

*CI* confidence interval, *RR* risk ratio

^a^Estimated via Longitudinal Targeted Maximum Likelihood Estimation with baseline and time-varying covariates. Baseline covariates included sex, race/ethnicity, education, household income, serving as uniformed workers during the 9/11 disaster, serving as rescue/recovery/cleanup workers at World Trade Center, Staten Island, and/or barge sites, and developing probable PTSD. Time-varying covariates included age, current smoking, marital status, employment status, and PTSD at Waves 2 and 3. In addition, social integration, physical health needs, and mental health needs at Wave 3 were included to account for intermediary paths through changes in the outcome

**Table 5:** Natural direct and indirect effects of frequent residential moving on depression through mediators among World Trade Center Health Registry Wave 4 Survey Participants, New York, 2003–2016

	High social integration (*N* = 21,376)		Unmet MH care (*N* = 4565)		Unmet medical care (*N* = 13,735)	
	OR	95% CI	OR	95% CI	OR	95% CI
Natural direct effect						
Stay–Stay (reference)	–	–	–	–	–	–
Move–Move	1.22	1.02, 1.47	1.01	0.78, 1.31	1.24	1.00, 1.53
Move–Stay	1.05	0.93, 1.19	0.95	0.79, 1.15	1.03	0.89, 1.19
Stay–Move	1.10	0.95, 1.26	1.04	0.85, 1.29	1.10	0.94, 1.30
Natural indirect effect						
Stay–Stay (reference)	–	–	–	–	–	–
Move–Move	0.93	0.90, 0.97	1.02	1.00, 1.05	0.99	0.97, 1.01
Move–Stay	0.98	0.95, 1.00	0.98	0.96, 1.00	1.00	0.99, 1.01
Stay–Move	0.99	0.96, 1.02	1.01	0.99, 1.03	1.01	0.99, 1.02

Causal mediation analysis was restricted to Wave 4 survey participants with complete data. The sample size differed across three mediation analyses because of difference in missing data across three mediators. In causal mediation analysis, we used baseline covariates (sex, race/ethnicity, education, household income, serving as uniformed workers during the 9/11 disaster, serving as rescue/recovery/cleanup workers at World Trade Center, Staten Island, and/or barge sites, and developing probable PTSD) and Wave 3 covariates (age, current smoking, marital status, employment status, and PTSD)

Type III ANOVA concluded that overall natural indirect effects through social integration (*p*-value = 0.008) and unmet mental health needs (*p*-value = 0.015) were statistically significant at two-sided *p*-value < 0.05

## Discussion

We found that frequent residential moving compared with stable residential status over 8 years was associated with risk of depression among adults who were exposed to the 9/11 disaster; however, infrequent moving (Move–Stay, Stay–Move) was not associated with risk of depression. In addition, we found evidence that social integration could alleviate higher risk of depression among frequently moving enrollees, pointing to a potential mechanism that explains a relationship between frequent residential moving and depression.

It has been well documented that housing instability is associated with a wide range of negative health outcomes [19]. Multiple studies demonstrate that displacement due to natural disasters [20], conflicts [21], or gentrification [22], is followed by increased stress and other adverse mental health conditions. However, thus far it has been unclear whether these negative health impacts are due to a direct effect of housing instability or preexisting differences between movers and non-movers [23, 24]. Utilizing rich longitudinal data from a large cohort and an advanced causal inference method that allows for controlling for bias due to missing data and time-varying confounding, the present study demonstrates that moving itself does negatively impact mental health. This suggests that residential instability, beyond baseline characteristics (e.g., low socioeconomic status and minority status), uniquely contributes to health disparities, and supports policies or programs that promote housing stability for their health benefits.

The mechanism through which social integration impacts health is well supported by Berkman and colleagues’ theoretical discussions [25]. They have proposed the social integration and health framework wherein housing stability promotes health as an upstream factor that strengthens social networks and ultimately health [25]. Strong social networks allow for increased social capital and collective efficacy, in turn encouraging community members to share health-promoting knowledge and adopt healthy behaviors [26]. In contrast, frequent residential moving may disrupt social networks and increase the likelihood of social isolation, negatively impacting enrollees’ capacity to manage stress [27]. We believe that our finding strengthens the existing literature and provides important evidence to support the role of social networks in explaining a link between housing instability and mental health.

Unlike social integration, we found that unmet medical needs did not explain excess risk of depression among frequent movers. In other words, frequent moving does not appear to create additional barriers for seeking healthcare among WTCHR enrollees. Instead, symptom severity has been previously identified as a strong predictor of unmet mental healthcare needs among this cohort [28]. Increased educational efforts to inform 9/11 exposed enrollees of treatment programs could also explain the null finding in our study. In 2009, the WTCHR created a Treatment Referral Program which conducted outreach encouraging 9/11 survivors to seek specialized care for their physical and mental health needs. This program was developed in response to the low utilization of healthcare treatment observed among 9/11 survivors. Staff was trained in motivational interviewing to help educate enrollees on the possible connection between WTC exposure and their symptoms, identify barriers to care, and link enrollees to 9/11 specialized care [29]. These outreach efforts may be resulting in initiating care or preserving continuity of care for enrollees who tend to move.

The current study has limitations. First, the study population came from individuals from one geographic area (i.e., New York City Metropolitan area) and were exposed to the 9/11 disaster. Given the unique background of the study population, a relationship between moving and depression may not be generalizable. Second, a formal causal mediation analysis approach has not been developed within a longitudinal TMLE framework to include in our study. To address this limitation, we examined indirect pathways via unmet healthcare needs and social integration using the modified exposure variable (fixed exposure vs. dynamic exposure) and a subset of the cohort (Wave 4 participants with non-missing data). Third, bias due to unobserved confounding cannot be ruled out and results can be biased either toward the null or away from the null, although a large number of time-invariant and time-varying covariates were included in the model. Fourth, the study did not collect data on reasons to move or secular trends that could uniquely impact mental health (e.g., the 2008 financial crisis), limiting the possibility of contextualizing a relationship between frequent residential moving and depression and generalizing findings beyond the WTCHR enrollees. Despite these limitations, several strengths should be noted. First, TMLE efficiently controlled for bias due to confounding and missing data in longitudinal data and drawing causal inference on the dynamic impacts of residential moving on depression. Second, we used administrative data to create an objective measure of residential movement over 8 years, strengthening the validity of the study findings.

## Conclusion

In this large cohort of individuals exposed to the 9/11 disaster, 6% have moved at least once every 4 years during the 8-year period, and experienced higher risk of depression. However, even after controlling for baseline and time-varying characteristics, those who moved only during the first or second 4 year periods (26%), as opposed to those who did not move (68%), did not have increased risk of depression. For a subset of the cohort that consisted of the Wave 4 survey participants with the complete data, social integration mediated a frequent residential moving—depression pathway. These findings highlight the importance of social networks in understanding how housing stability shapes risk for depression. For individuals experiencing frequent moving, especially those exposed to traumatic events, support for social connection to new communities should be considered an effective preventive measure against depression.

## Appendix

See Fig. 2 and Table 6.

**Table 6:** Unmet Health Needs/Social Integration outcomes (%) by residential moving patterns

	All		Stay–Stay		Move–Move		Move–Stay		Stay–Move	
	*N* ^a^	%^b^	*N* ^a^	%^b^	*N* ^a^	%^b^	*N* ^a^	%^b^	*N* ^a^	%^b^
Unmet physical health needs at Wave 3	28,354	8	19,679	8	1550	12	4082	10	3043	10
Unmet physical health needs at Wave 4	14,149	3	9686	2	803	3	2163	3	1497	4
Unmet mental health needs at Wave 3	28,223	12	19,577	10	1197	17	4070	15	3034	14
Unmet mental health needs at Wave 4	4679	29	2888	29	375	34	829	24	587	30
High social integration at Wave3	28,354	58	19,685	58	1547	54	4077	58	3045	55
High social integration at Wave4	21,977	58	15,251	59	1176	59	3269	59	2281	57

Sample sizes varied by variables because unmet physical and mental health needs were measured only among individuals who reported needs for physical and mental health cares

^a^Denominator

^b^% of individuals with outcomes

## Abbreviations

PTSD: Post-traumatic stress disorder
PUMA: Public use microdata areas
TMLE: Targeted maximum likelihood estimation
WTC: World Trade Center
WTCHR: World Trade Center Health Registry
